# Adverse events related to total ankle replacement devices: an analysis of reports to the United States Food and Drug Administration

**DOI:** 10.1007/s00264-021-04972-z

**Published:** 2021-02-11

**Authors:** Karim Mahmoud, Sreenivasulu Metikala, Kathryn M. O’Connor, Daniel C. Farber

**Affiliations:** Department of Orthopaedic Surgery, University of Pennsylvania Perelman School of Medicine, Philadelphia, PA USA

**Keywords:** Total ankle replacement, Adverse event, Device failure, MAUDE database

## Abstract

**Background:**

The published outcomes of total ankle replacement (TAR) implants came from limited institutions creating observational bias. For broader perspective, we queried the Food and Drug Administration’s (FDA) Manufacturer and User Facility Device Experience (MAUDE) voluntary database to explore complications reported outside published literature.

**Methods:**

The database was reviewed retrospectively between November 2011 and April 2019 using two product codes assigned to six TAR devices.

**Results:**

Among 648 relevant reports available in the database, common complications were aseptic loosening (19.3%), infection (18.2%), and alignment/mechanical issues (16.5%). Others included instrument/instrumentation complications, impingement, polyethylene problems, fractures, avascular necrosis of talus (AVN), and packaging issues.

**Conclusion:**

MAUDE database revealed various patterns of device-related malfunctions that have been under-reported in published data. Despite inconsistency in the available reports, it provided opportunities for improvements in quality control, device design, and ultimately patient safety. Database would be further strengthened by more robust reporting mechanism or mandatory reporting of device-related complications.

## Introduction

Total ankle replacement (TAR) has gained wide acceptance and popularity since its introduction in the early 1970s [1, 2]. Although ankle arthrodesis has been the gold standard for the management of end-stage ankle arthritis, it has the disadvantages of loss of motion at tibiotalar joint and potential risk of development of adjacent joint arthritis [3, 4]. Studies on TAR reported superior gait patterns [5–9] with preservation of motion of adjacent joints [10]. Given these reasons, the proportion of ankle arthroplasty procedures has been increasing substantially every year [11, 12] and TAR has become an attractive alternative to arthrodesis in select cases. Likewise, the rates of complications and revisions are also on the rise which appear to be greater than that of total hip and knee arthroplasty implant systems [13, 14]. However, most of the published data on the problems related to TAR devices came from individual case series that were reported by a limited number of health care facilities and surgeons. Hence, the statistics described in the publications are unlikely to reflect all the current modes of the adverse events across the national community of surgeons performing TARs and may not represent the experience of lower-volume surgeons.

In an effort to widely explore the complications related to TAR implants across the USA, we queried the USFDA’s Manufacturer and User Facility Device Experience (MAUDE) database, a voluntary platform designed to report malfunctions of medical devices [15]. Using a three letter code assigned to each device in the database, the data on the available adverse events can be sorted by the year of surgery or manufacturer to select the reports of interest. This data is publicly available with no attached personal health information. We hypothesized that a review of the MAUDE database would be an effective method to isolate different problems related to TAR devices that may not have been reported in the published literature. A similar method has been previously implemented exploring adverse events of shoulder arthroplasty implants as well as total elbow replacement and radial head arthroplasty [16, 17]. To our knowledge, the MAUDE database has not been previously researched to ascertain the characteristics of TAR complications.

## Material and methods

Retrospective investigation of the MAUDE database [18] was performed between November 1, 2011, and April 30, 2019, to survey the reports on TAR adverse events. A manual search of the FDA’s published Device Classification list detected 2 codes: NTG and HSN that were assigned to seven TAR prostheses including Scandinavian Total Ankle Replacement (STAR- Stryker, Kalamazoo, MI), Trabecular Metal Total Ankle (TMTA - Zimmer, Warsaw, IN), Vantage TAR system (Exactech, Gainesville, FL), Infinity/Inbone (Wright Medical Technology, Memphis, TN), Salto Talaris (Integra, Plainsboro, NJ), Cadence (Integra, Austin, TX), and Agility (DePuy, Warsaw, IN). All the available reports were evaluated to determine the total number of adverse events, types of adverse events, and any individual issues that were unique to a particular device.

## Results

**Fig. 1:**
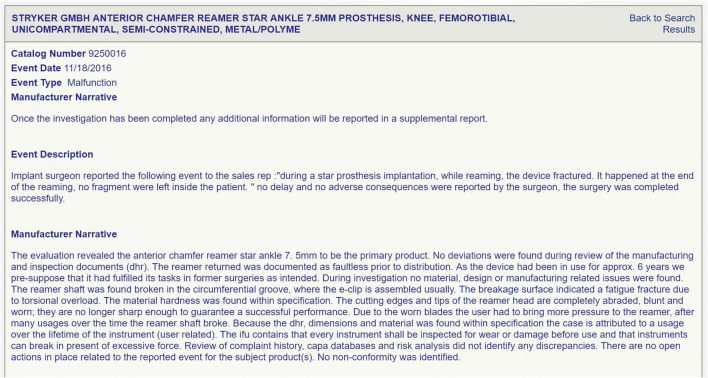
Example of an adverse report with the catalog number, event date, and type followed by a brief description of the event and manufacturer narration in the MAUDE database indicating a fracture of a reaming instrument

**Fig. 2:**
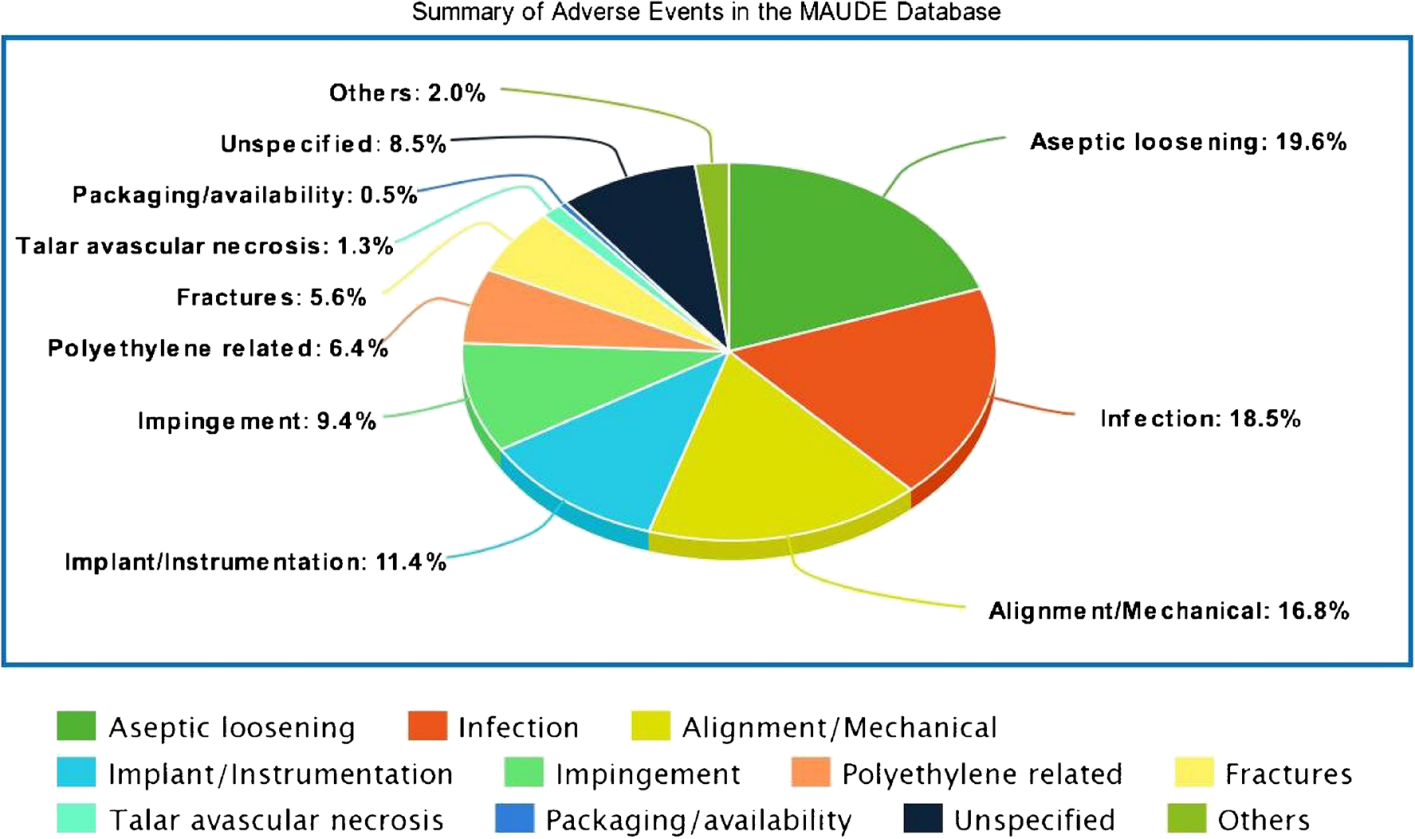
Summary of adverse events reported on MAUDE database

A total of 993 reports were detected in the MAUDE database during the aforementioned study period. After the exclusion of duplicate reports and non-arthroplasty reports, 648 specific events remained for final analysis. Each report typically has the model/catalog number, device problem, event date, and type followed by a brief description of the event and manufacturer narration (Fig. 1). Since the terminology used by the reporters for describing the problems was highly variable, every effort has been made by the authors to categorize all 648 adverse events into meaningful subgroups as depicted in Table 1 and Fig. 2.

**Table 1:** Assigned subgroups for the different terms of adverse reports mentioned in the MAUDE database

Assigned subgroups	MAUDE terminology
Aseptic loosening	Severe osteolysis
	Loose implant
	Subsidence
Infection	Superficial infection
	Deep infection
	Deep sepsis
Alignment/mechanical issues	Instability
	Malalignment
	Malposition
Implant/instrumentation problems	Pin/screw/drill/reamer breakage
	Tibial stem breakage
	Implant dislodgement
	Alignment guide problems
	Custom jig problems
Impingement	Soft tissue impingement
	Bony impingement
	Heterotopic ossification
	Gutter tightness
Polyethylene-related problems	Loose poly
	Small/large poly
	Fractured poly
	Poly dislodgement
	Poly wear
Fractures	Fracture of medial malleolus
	Fracture of lateral malleolus
	Periprosthetic fractures of tibia/talus
Packaging/availability problems	Package-partially opened
	Expired date
	Late arrival of inventory

**Fig. 3:**
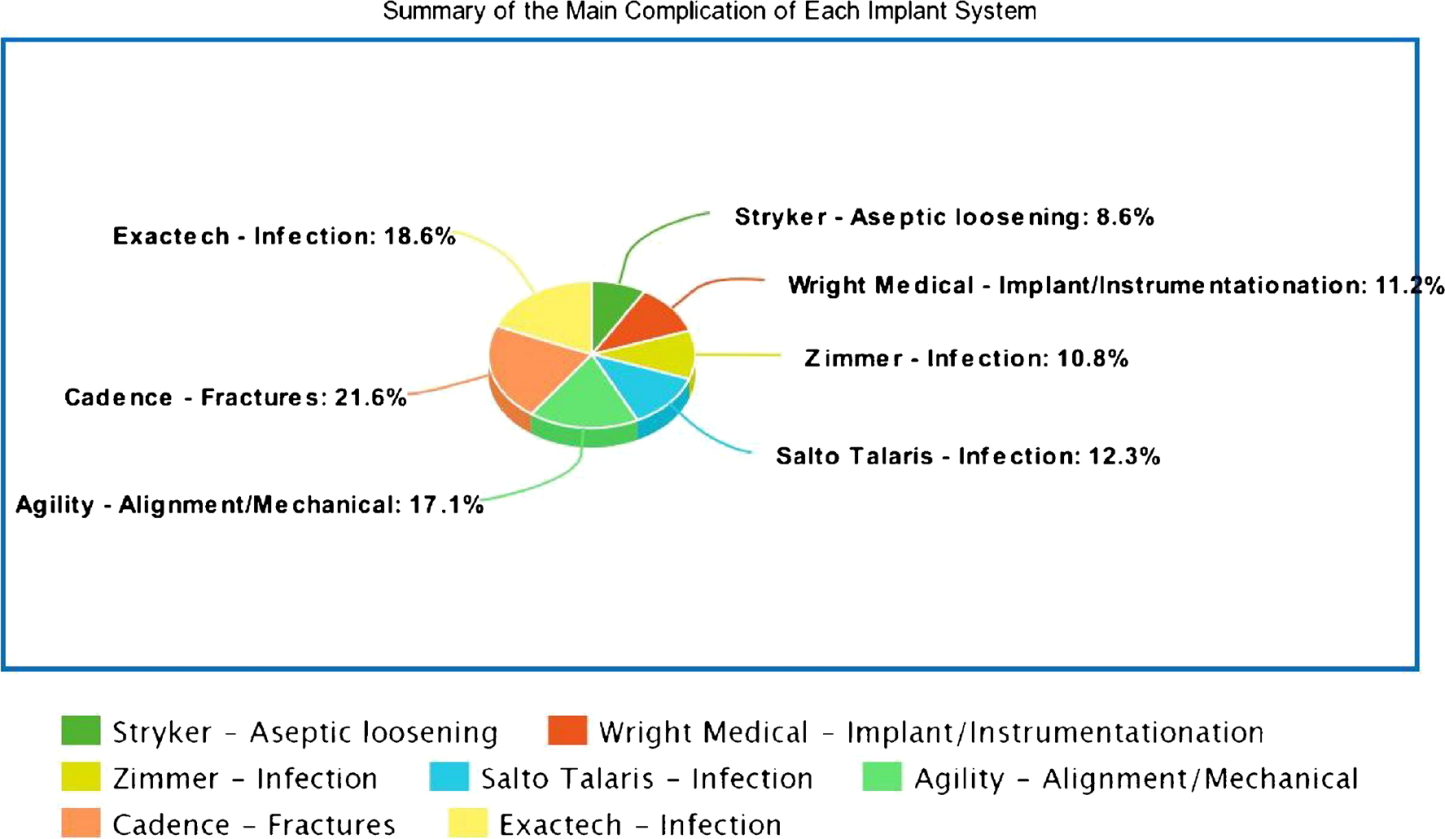
Summary of the main complication for each TAR system reported on the MAUDE database

Furthermore, the data has been summarized in Table 2 and Fig. 3 according to each TAR device. The most common reported event was aseptic loosening (19.3%) followed by infection (18.2%) and alignment/mechanical issues (16.5%). Other reports included instrument/instrumentation issues (11.26%), impingement problems (9.26%), polyethylene-related problems (6.32%), fractures (5.56%), AVN of talus at time of explant (1.23%), and packaging issues (0.46%). Incomplete reports with no available data to ascertain specific complications or adverse events were grouped as unspecified events which represented 8.33% of all reports. Two incidents of pulmonary embolism and one each of peroneal tendinosis, tibialis posterior tendinosis, allergic reaction, superficial peroneal nerve palsy, amputation, and death were also mentioned in the database. The complications of distal fibular fixation such as four reports of nonunion and one report of plate breakage were described only for the Zimmer Trabecular Metal TAR system.

**Table 2:** Summary of adverse events in the MAUDE database according to subgroups and TAR devices

Subgroups	Stryker	Wright Medical	Zimmer	Salto Talaris	Agility	Cadence	Exactech	Subtotal
Aseptic loosening	83	21	9	11	1	n/a		125 (19.6%)
Infection	56	16	22	17	n/a	3	4	118 (18.5%)
Alignment/mechanical	66	16	13	3	6	1	2	107 (16.8%)
Implant/instrumentation	32	34	4	1	1	1	n/a	73 (11.4%)
Impingement	33	2	12	12	1	n/a	n/a	60 (9.4%)
Polyethylene related	40	n/a	n/a	n/a	n/a	n/a	1	41 (6.4%)
Fractures	19	4	2	3	n/a	7	1	36 (5.6%)
Avascular necrosis-Talus	7	n/a	1	n/a	n/a	n/a		8 (1.3%)
Packaging/availability	2	n/a	1	n/a	n/a	n/a		3 (0.5%)
Others	Posterior tibial-tendinosis (1) SPN palsy (1) PE (2) Amputation (1) Death (1)	n/a	Peroneal tendinosis (1) Allergic reaction (1) Nonunion of fibula (4) Plate breakage (1)	n/a	n/a	n/a	n/a	13 (2%)
Unspecified	21	20	5	4	4	n/a	n/a	54 (8.5%)
Total reports	365	113	76	51	13	12	8	638

*n/a* not available, *PE* pulmonary embolism, *SPN* superficial peroneal nerve

## Discussion

The MAUDE database of the United States FDA is a publicly available online resource that is federally funded and continually updated with the voluntarily reported individual device-related problems. The database can readily be explored according to the product class or device name using the designated three letter code and further researched by the year and manufacturer to locate reports of interest. Unlike published literature that depicts the outcomes for a relatively limited group of surgeons, MAUDE data provides data regarding device-related malfunctions reported by a broad range of health care professionals including community and low volume practitioners. Besides, published data may be subject to observational or selection bias as reporting authors in many cases have helped to develop various prosthetic designs. Such a possibility is rare to occur in the MAUDE database. Among the 648 descriptions in the MAUDE website, 371 were identified for STAR device, 113 for Wright Medical implants, 80 for Zimmer TM, 51 for Salto Talaris, 13 for Agility, 12 for Cadence, and eight were for Exactech implants.

A striking difference can be seen among the TAR-related complications reported in the MAUDE data versus published literature. An analysis of recently published data [19] on the intermediate-term experience with STAR devices in the USA revealed 21 implant-related issues among 138 implantations at an average of five year follow-up. They consisted of alignment problems (38%), periprosthetic fractures (15.2%), polyethylene fractures (6.5%), impingement issues (3.6%), infection (2.9%), symptomatic cysts that required debridement (2.2%), and amputation (1.4%). Similar problems were identified in the MAUDE database in addition to 8.6% (*n* = 32) reports specifically related to dysfunction of instrumentation, and 1.9% (*n* = 7) cases of AVN of the talus which were not mentioned in the above publication.

Similarly, the first published report using the Infinity TAR system on clinical and radiographic outcomes of 67 patients [20] with a minimum of two year follow-up disclosed two cases (3%) each of aseptic loosening and heterotopic ossification along with one patient (1%) each with deep infection and intra-operative lateral malleolus fracture. Results of the INBONE I prosthesis [21] that completed a minimum of four to ten years of follow-up in a consecutive series of 149 patients at a single institution displayed 9.4% failures due to cysts/loosening (4.7%), talar subsidence (2.7%), and one (0.7%) each with fracture of the tibial component, chronic impingement, and infection. No reports of polyethylene problems, periprosthetic fractures, or instrument-related issues were described in the published data with either of the above devices. Conversely, 30% (*n* = 34) of the MAUDE adverse reports described for the above two Wright Medical TAR implants were attributed to the implant and custom jig instrumentation.

The published data on a single surgeon’s initial experience [22] of 55 primary total ankle arthroplasties using the Zimmer TMTA reported 93% implant survival at 24 months of follow-up. Their complications were three cases of aseptic loosening, two infections, three instances of impingement problems, and one case each of malalignment, intra-operative medial malleolar fracture, and talar fracture. Another case series of 16 patients using the same implant system with a two year follow-up revealed three cases of delayed/nonunion of fibular osteotomy [23]. Unlike other TAR devices, complications of distal fibular fixation were unique to the Zimmer Trabecular Metal TAR system since it requires a fibular osteotomy. Theoretically, this lateral transfibular approach has an advantage of a reduction in the potential for vascular injury to the talus [24], although one incident of talus AVN was specified in the MAUDE database which was, once again, never reported in the published data.

Likewise, a review of 72 patients that had Salto Talaris total ankle replacement with at least a five year follow-up demonstrated 95.8% survivorship [25]. Two incidents of aseptic loosening of the tibial component and one with chronic wound infection were mentioned as major complications requiring revision procedures. Other complications reported in this study were nine cases of impingements requiring gutter debridements, one with tarsal tunnel syndrome managed by open release and posterior tibial nerve repair, and one case of symptomatic periprosthetic bone cyst treated by bone grafting and polyethylene spacer exchange. No periprosthetic fractures were specified in this publication while three such reports were reported in the MAUDE database related to the same TAR implant.

To sum up, exploration of the MAUDE database had provided insight into the various intraoperative and postoperative problems associated with multiple TAR prostheses. However, it should be noted that there are several limitations to this study. First, the terminology of the available reports in the database was highly variable. This is due to the lack of a standard template for reporting a given adverse event which led to the inconsistency of submitted data. A similar problem exists in the published literature too. Glazebrook et al. recognized this issue and proposed a simplified classification system based on the severity of complications leading to TAR failure [26]. Later on, Mercer et al. described a detailed prototype worksheet for the standardized assessment and reporting of adverse events associated with TAR systems [27]. Such a system is not available in the MAUDE website. Secondly, the absolute rate of adverse incidents cannot be determined as the total number of TAR implantations of a given manufacturer during the study period was unknown. Also, the voluntary basis of the database reporting prevents the estimation of the true incidence rates. Few examples cited in the database including pulmonary embolism, neighboring tendon and nerve pathologies, allergic reaction, amputation, and death may not be pertinent to TAR devices. Finally, as with any online database, the accuracy and completeness of the details rely on the entity that reports it. Nevertheless, MAUDE data revealed several adverse mechanisms for every TAR prosthesis that have not been described in the published literature.

## Conclusions

This investigation of the MAUDE database provides insight into the spectrum of adverse events for total ankle arthroplasty compared to the published literature. The MAUDE website provides a convenient method to quickly report device-related issues by a broad range of health care professionals including community practitioners. However, the underutilization of this resource limits the utility of this information. More consistent or even mandatory reporting along with standardization of terminology would vastly improve the usefulness of this database in detecting implant problems and protecting the public.

## Data Availability

Not applicable
